# Towards an integrated neonatal brain and cardiac examination capability at 7 T: electromagnetic field simulations and early phantom experiments using an 8-channel dipole array

**DOI:** 10.1007/s10334-021-00988-z

**Published:** 2022-01-08

**Authors:** Jérémie Clément, Raphaël Tomi-Tricot, Shaihan J. Malik, Andrew Webb, Joseph V. Hajnal, Özlem Ipek

**Affiliations:** 1 Department of Biomedical Engineering, School of Biomedical Engineering and Imaging Sciences, King’s College London, London, UK; 2 MR Research Collaborations, Siemens Healthcare Limited, Frimley, UK; 3 Centre for the Developing Brain, King’s College London, London, UK; 4 Department of Radiology, C. J Gorter Center for High Field MRI, Leiden University Medical Center, Leiden, The Netherlands

**Keywords:** Neonate dipole coil array, Parallel-transmit, SAR reduction, Neonatal brain and heart, Ultra-high field, 7 T

## Abstract

**Objective:**

Neonatal brain and cardiac imaging would benefit from the increased signal-to-noise ratio levels at 7 T compared to lower field. Optimal performance might be achieved using purpose designed RF coil arrays. In this study, we introduce an 8-channel dipole array and investigate, using simulations, its RF performances for neonatal applications at 7 T.

**Methods:**

The 8-channel dipole array was designed and evaluated for neonatal brain/cardiac configurations in terms of SAR efficiency (ratio between transmit-field and maximum specific-absorption-rate level) using adjusted dielectric properties for neonate. A birdcage coil operating in circularly polarized mode was simulated for comparison. Validation of the simulation model was performed on phantom for the coil array.

**Results:**

The 8-channel dipole array demonstrated up to 46% higher SAR efficiency levels compared to the birdcage coil in neonatal configurations, as the specific-absorption-rate levels were alleviated. An averaged normalized root-mean-square-error of 6.7% was found between measured and simulated transmit field maps on phantom.

**Conclusion:**

The 8-channel dipole array design integrated for neonatal brain and cardiac MR was successfully demonstrated, in simulation with coverage of the baby and increased SAR efficiency levels compared to the birdcage. We conclude that the 8Tx-dipole array promises safe operating procedures for MR imaging of neonatal brain and heart at 7 T.

**Supplementary Information:**

The online version contains supplementary material available at 10.1007/s10334-021-00988-z.

## Introduction

Magnetic resonance imaging (MRI) of the developing brain is an active research topic [1] and has demonstrated the potential to detect, for example, early brain injuries in neonates that lead to adverse neurodevelopmental outcomes [2–5]. Besides, cardiac MR imaging methods can help to assess cardiovascular abnormalities in newborns [6, 7]. The studies are usually performed at 3 T [8, 9]. However, in the past years, there has been an increasing interest in 7 T MR as higher signal-to-noise ratio, contrast-to-noise ratio, and spatial resolution can be achieved [10–13]. These advantages associated with moving to a higher field strength could benefit neonatal imaging, as previously shown from 1.5 to 3 T [9]. Neonatal MR imaging is usually performed on 3 T MR scanners using the in-built body volume coil for transmit RF signal. At 7 T, integrating a body coil to the MR scanner is an ongoing research topic [14, 15] and is challenging as RF inhomogeneity is a limiting factor [10, 16]. Nevertheless, while an integrated RF coil could be well suited for whole-body adult imaging, the small size of the neonatal body may be spatially covered with local RF coils, as currently done for distinct adult body parts [17–20]. As an example, the birdcage coil [21] has been extensively used for adult brain MR studies [22–24] and may be used for neonatal brain MR imaging.

At the same time, RF safety is a critical aspect when performing neonatal MRI. RF power deposition in tissue is increased at 7 T compared to lower fields as a result of the increased frequency of the electromagnetic wave [25, 26]. On another side, when moving from adult to baby, although power deposition was decreased at 1.5 T and 3 T using the large body coil [26] there is no clear evidence that the same observation applies at 7 T, when using local transmit coils [27, 28]. Specific-absorption-rate (SAR) quantifies the power deposition when the subjects are exposed to RF fields [29]. A conventional birdcage coil is typically fed through two ports driven in circularly polarized mode from a single RF power source. Therefore, the SAR levels are straightforward to derive [27] as the RF field distribution is fixed for a given load, but this gives no flexibility to control SAR, which may mean the maximum RF power has to be reduced, in turn impairing image quality.

The parallel-transmit approach is known to increase the flexibility of RF transmission at high fields. Single RF coil elements are combined into multi-channel arrays and geometrically distributed around a region-of-interest. Transmit arrays were initially proposed to tackle RF inhomogeneity at ultra-high field by providing additional degrees of freedom for shimming the RF field distribution pattern [30, 31]. In addition, the individual RF phases may be optimized to simultaneously reduce the SAR levels in the neonatal body as previously reported for adult cases [32, 33]. However, it is unclear how RF shimming performs in neonatal brain and heart. These two organs are located at different depths and have different sizes but since they are significantly smaller compared to the adult, the RF inhomogeneity typically observed at 7 T [16] may be lower and B_1_^+^-field efficiency could be increased as the dissipative volume is smaller. Therefore, it may be easier to cover homogeneously either the baby’s brain or heart without modification of the coil array design.

Radio-frequency coil arrays using dipoles as transmit elements have been previously reported and extensively used for adult brain or body MR applications at 7 T [19, 34–37] and were compared to loop coils [38], but were so far not investigated for neonatal applications. Following the established literature, the centre-shortened dipole could be particularly suited as it is straightforward to implement and demonstrated good RF performances compared to other dipole design approaches, as fractionated dipole arrays, on the adult brain [34]. However, to our knowledge, the dipole arrays reported so far were designed such that the elements are placed either directly on the subject for cardiac or prostate imaging [36, 39], or close to the subject for brain imaging [34, 40]: proximity maximizes the power flux towards the conductive tissues and provides high B_1_^+^-field efficiency [38]. Fitting the entire neonate together with adapted bed support necessarily requires the RF elements to be placed at a relatively large distance from the body. There is no clear evidence on how the dipoles will perform in such situation, notably in terms of RF performance.

Therefore, this study aimed to introduce a whole-body 8-channel dipole array for neonatal brain and heart MR applications. The B_1_^+^-field efficiency and SAR levels are evaluated to estimate the combined effects of subject size (adult vs baby), and target size/location (brain vs heart) on the RF performance of the coil array in comparison with a single transmit birdcage coil at 7 T.

## Methods

### RF coil array design

**Fig. 1:**
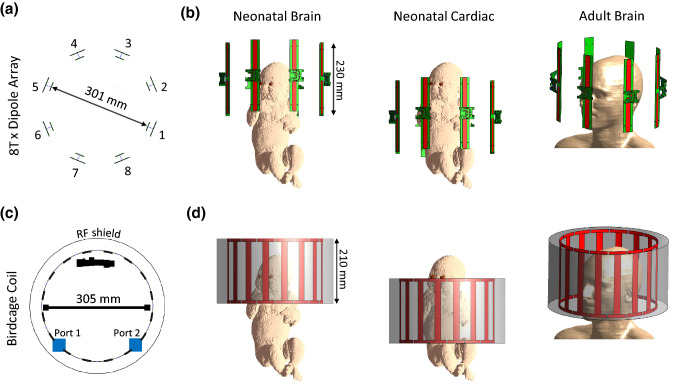
RF coil dimensions and simulated models for **a** and **b** the 8Tx-dipole array and **c** and **d** the 16-legs birdcage coil. The coils are shown in neonatal brain, neonatal cardiac, and adult brain configurations (**b** and **d**). For the birdcage coil, the two ports were placed at the top of the coil and driven in quadrature. For the 8Tx-dipole array, the feed cables for dipoles were directly soldered to their centre junction and passed out of the coil outer housing (not shown) through a small hole at the head end of the structure. Cables were not included in either model for the simulations

**Fig. 2:**
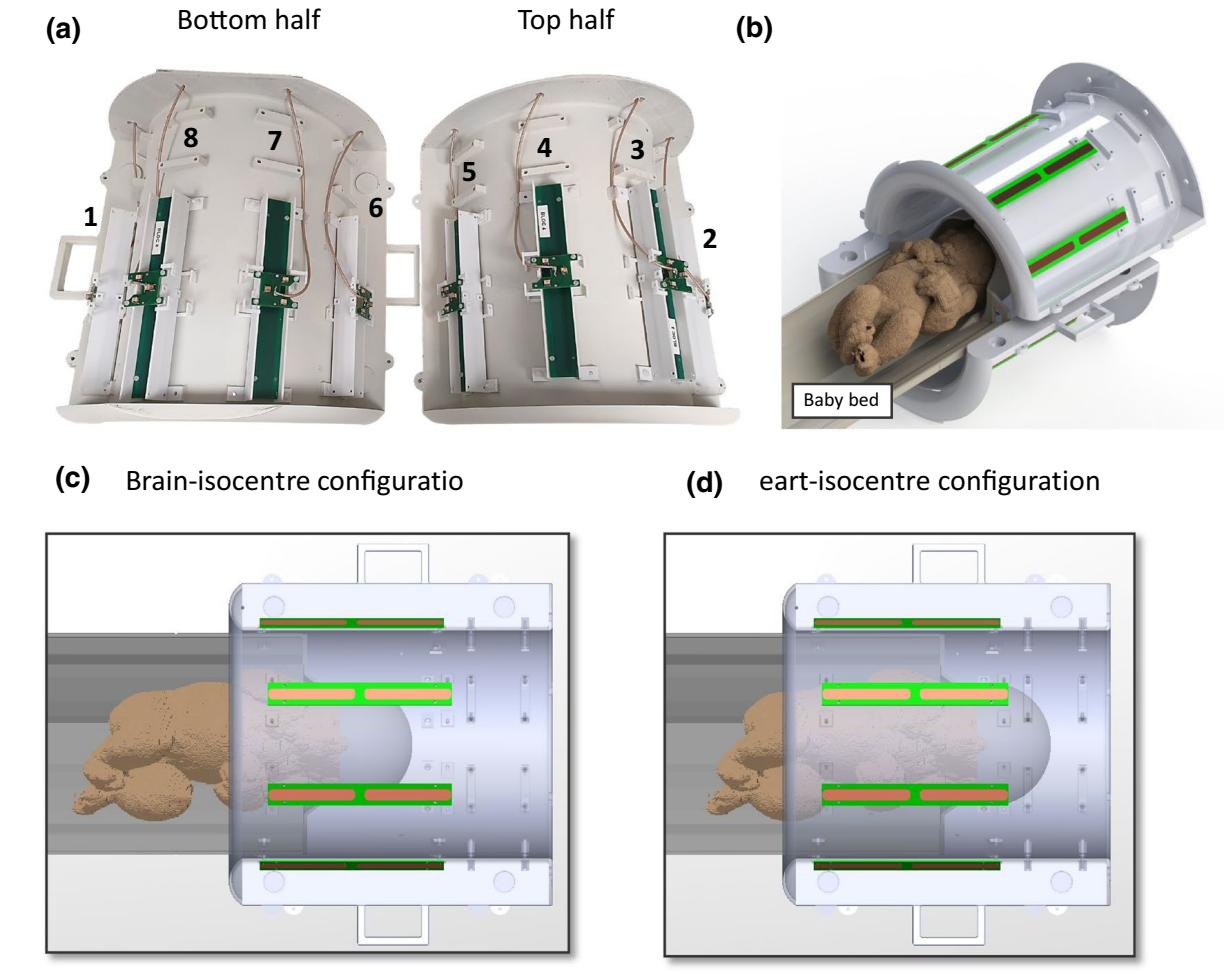
**a** Photo of the in-house built 8Tx-dipole array when the coil array is separated in two halves. **b** 3D render of the neonatal setup showing the splitting with the baby model in-place. **c** and **d** 3D render of the neonatal setup with the baby model in-place, in **c** the brain-isocentre and **d** the heart-isocentre configurations. The coil holder structure was made transparent to better visualize the neonatal bed fitting (in dark colour for better contrast)

The 8-channel dipole array (8Tx-dipole array) for neonatal brain and cardiac MR applications consisted of eight centre-shortened dipole antennas (width = 15 mm) with each dipole etched from 35 µm copper on a 1.6 mm-thick FR-4 substrate (Eurocircuits LTD, United Kingdom). Dipoles were placed at 45° from each other on a cylinder with a large diameter of 301 mm (Fig. 1a) as it accounted not only for the whole-body size of neonates but also for the minimal required space to install a dedicated bed (Fig. 2). A dipole length of 230 mm, significantly larger than the neonatal heart and brain dimensions (50 to 90 mm in head-foot direction), was chosen to ensure the full longitudinal spatial coverage of the two organs and to get sufficient B_1_^+^-field efficiency as the depth profile of the transmit field intrinsically scales with dipole’s length [38]. Long dipoles ($\ge$ 150 mm) also tend to produce lower SAR maxima as the current is more evenly distributed compared to small dipoles [38], which was a critical design criterion for neonatal applications. The centre position along the longitudinal axis for each pair of left–right symmetrical dipoles was moved up or down to account for the human brain geometry. The maximal shift distance was 25 mm, between dipoles 3–4 and 7–8 (Fig. 1b). The tuning/matching circuit consisted of two hand-wounded copper-wire series inductors, two series and one parallel capacitor (American Technical Ceramics, USA) placed on a printed-circuit board (same FR-4 substrate as the dipoles) elevated by 15 mm with respect to the dipole legs’ level, and symmetrically positioned with respect to them [34]. Dipoles were tuned and matched using a 4-channel vector network analyser (Keysight Technologies E5080A-ENA, USA).

A phantom was designed according to realistic neonate dimensions and filled with a saline solution at a concentration of 5.8 g/L (Fig. 8a) to closely match the average conductivity of the simulated neonate model (Table 1). The total volume was approximately 4.8L and the dielectric properties (*ɛ*_r_ = 79, *σ* = 0.95 S/m) were measured using a dielectric assessment kit (DAK 12, SPEAG, Switzerland).

**Table 1:** Adjusted dielectric properties (mass density, conductivity, and relative permittivity) used for the neonate model as published by Malik et al. [28]

Tissues/properties	Mass density [kg/m^3^]	Conductivity [S/m]	Relative permittivity *ɛ*_r_
Brain	1046	1.62	83.2
Heart	1081	1.35	90.3
Muscle	1090	1.30	80.9
Lungs (inflated)	394	0.53	32.2
Bone	1908	0.32	29.6
Thyroid Gland	1050	1.28	81.2
Fat	911	0.11	15.3
Eyes	1005	1.52	69.0
Gut	1030	0.77	58.2
Skin	1109	1.38	94.4
Blood (Aorta, Inferior vena cava)	1050	1.32	65.7
Liver	1079	0.80	61.1

A coil holder structure was drawn (Solidworks, Dassault Systèmes, France), 3D-printed in polycarbonate (Deed3D Technology Co., China), and painted with a lacquer (TOY Brand, the China Paint MFG. Co.) meeting the international standards (as ISO8124-3 and EU2005/84/EC) in terms of lack of toxicity. The MR transparency of the polycarbonate used was assessed, in the MR scanner, using standard imaging sequences as turbo-spin-echo (1.6 mm-iso, TR/TE = 2000/159 ms, FOV = 490 × 490 mm^2^) and MP2RAGE [24] (0.75 mm-iso, TR/TE = 4300/2.27 ms, FOV = 240 × 240 mm^2^). The coil holder was designed to be separated into two halves to facilitate the positioning of neonates for the MRI scans (Fig. 2a, b). The setup, including the neonate’s bed, is shown in Fig. 2c, d for the neonatal brain and cardiac configurations, respectively. The minimal distance between the neonate’s bed and the coil holder structure was about 2 mm. The same printing and painting approach as for the coil holder was used for the neonate’s bed.

### Electromagnetic field simulations

The electromagnetic field simulations were performed using a finite-difference time-domain (FDTD) simulation software (Sim4life 6.2, ZMT, Switzerland). The neonate model [28] was developed in-house and included 13 tissues which were segmented from in-vivo MR images to reflect the natural position of a baby undergoing an MR examination. Dielectric properties of all tissues were defined according to the adjusted values for the neonate model (Table 1) [28]. The coil holder was imported to place accurately the dipoles but not itself simulated, since it does not disturb the RF signal. The centre-shortened dipoles were modelled, including the FR-4 substrate (*ɛ*_r_ = 4, zero electrical conductivity), and conductive parts were defined as lossy metal (*σ* = 5.8e7 S/m). The magnet RF screen was added to the simulation model and defined as perfect electric conductor (PEC). Three simulation setups were carried out, neonatal brain and heart at the centre of the coil array, and brain-centred with the Duke adult human model (Fig. 1b) [41].

The baby and Duke models were gridded at 2 mm-isotropic resolution for resulting total grid sizes of 38 Mcells and 62 Mcells, respectively. The tuning/matching circuit layout included all the lumped elements used in the built array at their exact position for a proper correspondence between simulations and measurements. All RF ports (8 sources and 40 lumped elements) were driven individually by a Gaussian excitation centred at 297.2 MHz with a 200 MHz bandwidth for 300 periods with auto-termination when the convergence reaches to -50 dB. Computations were carried out on dedicated GPUs (2 × GTX Titan X, Nvidia Corp., USA). Convergence was usually achieved within 60 periods in $\sim$ 85 min per port. A co-simulation approach (Optenni Ltd, Finland) was used to adjust the lumped elements of the dipoles to tune and match at 297.2 MHz and 50 Ohms. The values were optimized with respect to the built array (50–70 nH for inductors, 1–22 pF for capacitors), and the final simulated values were similar to the constructed array. The process was done separately for neonatal and adult configurations, but the lumped-element values optimized for neonatal brain were applied in the neonatal cardiac position to mimic the real-case situation where no adjustment would be done between neonatal brain and cardiac imaging. For simulations with the neonate phantom, the same lumped-element values were used. Inter-element couplings and reflection coefficients were computed with the scattering matrices with the baby model. The simulated CAD model of the 8Tx-dipole array with phantom is available to download as supplementary data of this manuscript. In addition, to further evaluate the efficiency of the 8Tx-dipole array for neonatal MR applications in the given design configuration, an 8Tx-loop coil array was modelled and simulated with identical design criteria (Supporting Information).

### Evaluation of the simulated data

Simulated individual complex B_1_^+^-field maps and electric E-fields were interpolated at 1 mm-isotropic, normalized to 1 W total input power at the coil input, and exported to Matlab (R2021a, the MathWorks, Natick, MA). Q-matrices were derived from simulated E-field and tissue densities for 10 g-tissue mass-average regions and were used to evaluate SAR levels [42]. The worst-case SAR_10g_ (SAR_10g,wc_) is defined by the RF weights combination producing the highest possible SAR_10g_. A Q-matrix largest eigenvalue analysis was performed with the maximum eigenvalue $\left({\mathrm{SAR}}_{10\mathrm{g},\mathrm{max}}^{\mathrm{eigen}}\right)$ being the SAR_10g,wc_ when the total RF power is freely distributed across the channels [43]. In this case, the SAR_10g,wc_ value may be achieved when all the power goes into a single channel. The eigenvalue map describing the highest possible SAR_10g_ for each voxel and the eigenvector corresponding to the ${\mathrm{SAR}}_{10\mathrm{g},\mathrm{max}}^{\mathrm{eigen}}$ were computed to provide an insight into the dipoles producing the strongest contribution to the worst-case SAR levels. However, in parallel-transmit the maximum power per channel is limited, meaning that all the power cannot go into a single channel, while it is allowed with the eigenvalue approach. Therefore, the SAR_10g,wc_ was additionally assessed for 1 W total power equally distributed so that each channel is driven at full amplitude, and corresponding maximum intensity projection (MIP) maps were computed for the 8Tx-dipole array in the neonatal brain and cardiac positions. RF phases of individual channels were then optimized to maximize B_1_^+^-field in neonatal brain and heart regions while minimizing the overall SAR_10g,max_ value, using a particle-swarm algorithm [31, 44]. To accelerate the calculations, a set of Virtual Observation Points (VOP) was generated from the Q-matrices, following Eichfelder and Gebhardt [45], using manufacturer-provided compression software (Siemens Healthcare, Erlangen, Germany) with an overestimation coefficient of 10%. Equation 1 defines the SAR-optimized cost-function whose objective is to maximize the B_1_^+^-field over the region-of-interest while keeping a low SAR_10g,max_ value, and where N corresponds to the total number of pixels in the region-of-interest.

1 $$\mathrm{cost}= \sqrt{\frac{1}{\mathrm{N}}*\sum_{\mathrm{n }= 1}^{\mathrm{N}}{\left|{\mathrm{R}}_{\mathrm{n}}-{\mathrm{G}}_{\mathrm{n}}\right|}^{2}}$$

2 $${\mathrm{R}}_{\mathrm{n}}= \frac{{\left({\mathrm{SAR}}_{\mathrm{Eff}}^{\mathrm{MOS}}\right)}_{\mathrm{n}}}{{\left({\mathrm{SAR}}_{\mathrm{Eff}}^{\mathrm{SOM}}\right)}_{\mathrm{n}}}$$

3 $${\left({\mathrm{SAR}}_{\mathrm{Eff}}^{\mathrm{MOS}}\right)}_{\mathrm{n}}= \frac{{\left({\mathrm{B}}_{1,\mathrm{MOS}}^{+}\right)}_{\mathrm{n}} }{\sqrt{{\left({\mathrm{SAR}}_{10\mathrm{g},\mathrm{max}}\right)}_{\mathrm{VOPs}}}}\;\; \mathrm{and }\;\;{\left({\mathrm{SAR}}_{\mathrm{Eff}}^{\mathrm{SOM}}\right)}_{\mathrm{n}}= \frac{{\left({\mathrm{B}}_{1,\mathrm{SOM}}^{+}\right)}_{\mathrm{n}} }{\sqrt{{\mathrm{SAR}}_{10\mathrm{g}}^{*}}}$$

In Eq. 1, the unitless quantity *R*_n_, defined by Eq. 2, was calculated as the per-pixel ratio between the shimmed magnitude-of-sum (MOS) and the sum-of-magnitude (SOM) SAR efficiencies (Eq. 3). The shimmed SAR_10g,max_ value was calculated using the VOPs for the MOS SAR efficiency ${\left({\mathrm{SAR}}_{\mathrm{Eff}}^{\mathrm{MOS}}\right)}_{n}$. ${\left({\mathrm{B}}_{1,\mathrm{SOM}}^{+}\right)}_{n}$ over a fixed ${\mathrm{SAR}}_{10\mathrm{g}}^{*}$ value of 1 W/kg/W was chosen to compute ${\left({\mathrm{SAR}}_{\mathrm{Eff}}^{\mathrm{SOM}}\right)}_{n}$, where ${\left({\mathrm{B}}_{1,\mathrm{SOM}}^{+}\right)}_{n}$ represents an optimal solution as the spatial phase variations of the complex B_1_^+^-field for individual channels are eliminated. High values for the ${\left({\mathrm{B}}_{1,\mathrm{MOS}}^{+}\right)}_{n}$ combined with low SAR_10g,max_ value bring the ratio closer to 1, which means closer to the optimal solution characterized by ${\left({\mathrm{SAR}}_{\mathrm{Eff}}^{\mathrm{SOM}}\right)}_{n}$. The unitless quantity G_n_ represents the per-pixel value of a 2D-Gaussian pattern that has a unit value at the centre of the ROI, and the standard deviation values in the two directions defined such as out-centred *G*_n_ values are close but not equal to unit value of 1. G_n_ helps to prevent the algorithm to converge to specific shimmed solutions where high B_1_^+^-field is observed at the edges of the region-of-interest in a ring shape with a null at the centre. The SAR-optimized cost function (Eq. 1) computes the root-mean-square error between *R*_n_ and *G*_n_.

The B_1_^+^-field and SAR efficiency $\left({\mathrm{B}}_{1}^{+}/\sqrt{{\mathrm{SAR}}_{10\mathrm{g},\mathrm{max}}}\right)$ values were averaged over the whole-brain and whole-heart of the neonatal model and SAR_10g,max_ value was calculated over all tissues. For the adult head, the B_1_^+^-field was averaged over 5 brain tissues, white and grey matter, corpus callosum, cerebellum, and midbrain (*σ*_adult_ = 0.86 S/m; *ɛ*_r,adult_ = 53.5), which averaged conductivity and permittivity were different from the neonatal brain (*σ*_neonate_ = 1.62 S/m; *ɛ*_r,neonate_ = 83.2, Table 1). Note that the SAR_10g_ maps, and consequently the SAR_10g,max_ value was obtained using the full 10 g-averaged Q-matrices, before VOP compression, and applying the RF phases found using the SAR-optimized cost function (Eq. 1). The B_1_^+^-field and SAR_10g_ quantities were normalized to 1 W total input power.

The whole-body and head-average SAR values, defined as the ratio between the total absorbed power in the concerned tissue region (whole-body or head) and its total mass (Eq. 4), were also computed, for 1 W total input power, since a significant portion of the neonate is exposed to RF fields [46]. The total absorbed power in tissue was calculated with Eq. 5 where M is the total number of voxels, *E*_m_ and *J*_m_ are the per-voxel shimmed electric and current field values (with the bar denoting the complex conjugate), and *V*_m_ is the voxel’s volume.

4 $${\mathrm{SAR}}_{\mathrm{whole}-\mathrm{body}}= \frac{{\left({\mathrm{P}}_{\mathrm{absorbed}}\right)}_{\mathrm{body}}}{{\mathrm{Mass}}_{\mathrm{body}}}\;\mathrm{ and }\;{\mathrm{SAR}}_{\mathrm{head}}= \frac{{\left({\mathrm{P}}_{\mathrm{absorbed}}\right)}_{\mathrm{head}}}{{\mathrm{Mass}}_{\mathrm{head}}}$$

5 $${\mathrm{P}}_{\mathrm{absorbed}}= \frac{1}{2}* \left|\sum_{\mathrm{m}=1}^{\mathrm{M}}{\mathrm{E}}_{\mathrm{m}}* \overline{{\mathrm{J} }_{\mathrm{m}}}*{\mathrm{V}}_{\mathrm{m}}\right|$$

The head regions were manually defined, including head, neck, and shoulders for a total mass of 1.2 kg for the neonate model and the region was cropped right below the chin (mass ≈ 5 kg) for the adult model. The whole-body neonate mass was $\sim$ 3.7 kg.

As a comparison, all the aforementioned simulated quantities were computed for a 16-leg high-pass shielded birdcage coil, driven in circularly polarized (CP) mode, and whose dimensions (diameter = 305 mm, total length = 210 mm, rung/endring width = 20/10 mm, shield diameter = 372 mm) correspond to a commercially available adult head coil (Nova Medical Inc., MA, USA). The coil diameter was not modified although it would not fit the designed neonatal bed structure (Fig. 2c, d). The birdcage coil was tuned/matched for neonatal brain position, and lumped element values were kept the same for the neonatal cardiac position, as it was done for the dipole array. Matching to adult brain load was done independently. The three simulations performed with the 8Tx-dipole array—neonatal brain or heart at the centre of the coil, and adult brain-centred—were also carried out with the single-channel birdcage coil. Note that the birdcage coil design is shorter compared to the dipole array. To assess the validity of the simulated data, MR acquisitions were performed using the built phantom, and a 7 T MR scanner (MAGNETOM Terra, Siemens Healthcare, Erlangen, Germany) with 8 × 2 kW RF amplifiers in prototype research configuration. Individual B_1_^+^-field maps (magnitude/phase) were acquired per channel using a pre-saturation turbo-flash sequence [47], normalized to 1 kW total output power at RF amplifier and compared to the simulated B_1_^+^-field maps on the same phantom. The individual B_1_^+^ phase maps were computed relative to a shimmed mode. Simulated individual B_1_^+^-field maps were normalized to the corresponding averaged measured B_1_^+^-field value. The B_1_^+^-field distribution in circularly-polarized mode and for one RF shimmed case were measured with the actual-flip-angle method [48] and compared to the corresponding simulated B_1_^+^-field maps using the same RF phases. Both the CP and RF shimmed B_1_^+^-field maps were obtained with the total power equally distributed across each channel, and simulated maps were normalized to the central maximum B_1_^+^-field value of the measured maps. Difference maps were calculated, and the normalized root mean square error (NRMSE) was determined for magnitude B_1_^+^-field maps. The NRMSE was calculated as the root-mean-square error between simulated and measured B_1_^+^-field maps divided by the peak measured B_1_^+^-field value. To avoid isolated high B_1_^+^-field values that could result from measurement error, the peak was chosen as the 99th percentile value.

## Results

**Fig. 3:**
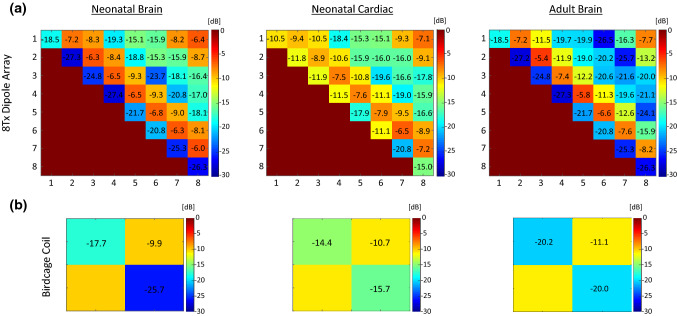
Simulated scattering matrices shown for **a** the 8Tx-dipole array and **b** the birdcage coil, and the three configurations shown in Fig. 1. The same parameters were used for neonatal brain and cardiac configurations. Tuning and matching of the dipoles and the birdcage coil for the adult brain was done separately

Figure 3 shows the scattering parameters for the 8Tx-dipole array and the birdcage coil for neonate brain and cardiac and adult brain imaging configurations. The reflection coefficients were lower than -18 dB for all the channels of the 8Tx-dipole array in the neonatal brain imaging position (Fig. 3a). When shifting to the cardiac centred position without recalibrating the coil for tuning and matching, the reflection coefficients were still better than − 10 dB while the average coupling value between closest neighbours was reduced by 20% (− 7.8 dB vs − 6.5 dB). The average nearest-neighbour coupling value of − 7 dB for the adult brain setup was higher compared to the neonatal cardiac configuration (− 7.8 dB) but lower compared to the neonatal brain configuration (− 6.5 dB). The coupling values for the birdcage coil were always near to or lower than − 10 dB and reflection coefficients were only slightly changed when shifting to the neonatal cardiac position, without re-tuning and matching (S_11_ >  − 14 dB).

**Fig. 4:**
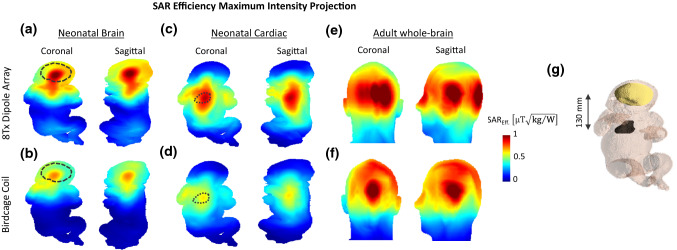
Maximum intensity projection of SAR efficiency (${\mathrm{B}}_{1}^{+}/\sqrt{{\mathrm{SAR}}_{10\mathrm{g},\mathrm{max}}}$) maps, in coronal and sagittal orientations for the 8Tx-dipole array (**a**, **c**, **e**) and the birdcage coil (**b**, **d**, **f**). Three different configurations, neonatal brain (**a**, **b**), neonatal cardiac (**c**, **d**) and adult brain (**e**, **f**) are shown. For the 8Tx-dipole array, RF phases were optimized over a ROI in a slice for SAR_10g,max_ reduction. Neonatal brain and heart positions are indicated as reference (black dashed-line region-of-interest). **g** Neonatal simulation model highlighting the brain and the heart. The distance between the two organs is indicated for reference

Complete spatial coverage of the neonatal brain and heart was demonstrated in SAR efficiency (${\mathrm{B}}_{1}^{+}/\sqrt{{\mathrm{SAR}}_{10\mathrm{g},\mathrm{max}}}$) maps for the 8Tx-dipole array and RF phases obtained using the SAR-optimized cost function (Fig. 4a,c and Eq. 1). The maximum intensity levels were localized at the organs’ positions, and the SAR efficiency distribution was longitudinally extended beyond the regions-of-interest. The averaged SAR efficiency values of 0.64 $\mathrm{\mu T}\sqrt{\mathrm{kg}/\mathrm{W}}$ (brain) and 0.73 $\mathrm{\mu T}\sqrt{\mathrm{kg}/\mathrm{W}}$ (heart) were higher in comparison with the CP mode as we applied B_1_^+^ and SAR optimisation in the region of interest (Table 2). In comparison with SAR-optimized maps for the dipole array, SAR efficiency was visibly lower for the birdcage coil in both neonatal brain and cardiac configurations. Moreover, although the brain and heart were spatially covered as the coil was longitudinally centred for those regions, the overall longitudinal coverage was lower for the birdcage coil (Fig. 4b, d) than for the 8Tx-dipole array (Fig. 4a, c) since their length in head-foot direction is different by design. The SAR_10g,max_ value after SAR-optimization was 85% and 62% lower with the 8Tx dipole array compared to the birdcage coil, in brain and cardiac configurations, respectively (Table 2). Figure 4e, f depicts the SAR efficiency maps as simulated on the adult head with the dipole array and the birdcage coil. As the volume-of-interest increased from 0.3L (neonatal brain volume) to 1.3L (total volume of the 5 adult brain tissues), the averaged SAR efficiency was decreased by 6% for the 8Tx-dipole array but increased by 22% for the birdcage coil (Table 2). The 8Tx-loop coil array achieved, in neonatal configurations, an averaged SAR efficiency value of 0.49 $\mathrm{\mu T}\sqrt{\mathrm{kg}/\mathrm{W}}$ (brain) and 0.60 $\mathrm{\mu T}\sqrt{\mathrm{kg}/\mathrm{W}}$ (heart) (Supporting Information Figure S1d).

**Table 2:** Quantitative results of the maps shown in Fig. 4 for the neonatal brain and cardiac and the adult brain configurations

	Transmit mode	Coil model	Mean B_1_^+^ over ROIs $\left[\mathrm{\mu T}/\sqrt{\mathrm{W}}\right]$	SAR efficiency $\left[\mathrm{\mu T}\sqrt{\mathrm{kg}/\mathrm{W}}\right]$	SAR_10g,max_ $\left[\mathrm{W}/\mathrm{kg}/\mathrm{W}\right]$	Whole-body average SAR $\left[\mathrm{W}/\mathrm{kg}/\mathrm{W}\right]$	Head-Average SAR $\left[\mathrm{W}/\mathrm{kg}/\mathrm{W}\right]$	Absorbed power [%]
Neonatal Brain	SAR optimized	8Tx Dipole Array	0.32 ± 0.06	0.64 ± 0.12	0.25	0.07	0.13	24
	CP mode	8Tx Dipole Array	0.34 ± 0.06	0.59 ± 0.10	0.33	0.08	0.15	29
		Birdcage Coil	0.60 ± 0.13	0.46 ± 0.10	1.68	0.22	0.53	82
Neonatal Cardiac	SAR optimized	8Tx Dipole Array	0.61 ± 0.11	0.73 ± 0.13	0.69	0.18	0.16	66
	CP mode	8Tx Dipole Array	0.60 ± 0.09	0.62 ± 0.09	0.94	0.17	0.14	61
		Birdcage Coil	0.67 ± 0.14	0.50 ± 0.10	1.82	0.25	0.24	91
Adult Brain	SAR optimized	8Tx Dipole Array	0.26 ± 0.08	0.60 ± 0.18	0.19	–	0.08	38
	CP mode	8Tx Dipole Array	0.26 ± 0.07	0.54 ± 0.15	0.23	–	0.07	37
		Birdcage Coil	0.39 ± 0.11	0.56 ± 0.16	0.49	–	0.15	75

The eight columns display the transmit mode, coil model, mean B_1_^+^ over the region-of-interest, the mean SAR efficiency computed as ${\mathrm{B}}_{1,\mathrm{mean}}^{+}/\sqrt{{\mathrm{SAR}}_{10\mathrm{g},\mathrm{max}}}$, SAR_10g,max_ value, the whole-body/head average SAR and the total absorbed power in tissue. All the quantities were normalized to 1 W input power. CP mode is indicated as a comparison for the 8Tx-dipole array. For the adult brain, the B_1_^+^ values were averaged over 5 brain tissues, white and grey matter, corpus callosum, cerebellum and midbrain

**Fig. 5:**
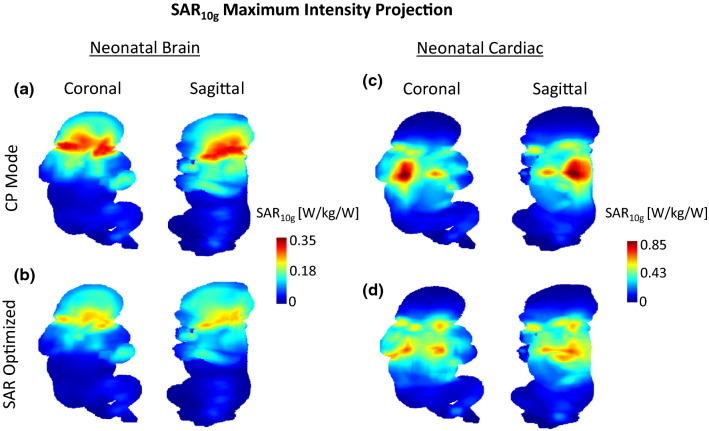
Maximum intensity projection of SAR_10g_ maps, in coronal and sagittal orientations for the 8Tx-dipole array. Two different configurations, neonatal brain (**a**, **b**) and neonatal cardiac (**c**, **d**) are shown for two distinct RF shimming weights. In (**a**, **c**), RF phases are defined for CP mode ($\mathrm{\Delta \phi }=45^\circ$ between each dipole). In (**b**, **d**) RF phases were optimized for SAR_10g,max_ reduction and are identical to Fig. 4a, c

A flattening of the SAR_10g_ distribution in neonatal brain and cardiac configurations was observed for SAR-optimized RF phases (Fig. 5b, d) with respect to the CP mode (Fig. 5a, c). While in CP mode the high SAR_10g_ levels were spatially concentrated at the edges of the model, they were visually more distributed after SAR optimization. This observation is consistent with the decrease of the SAR_10g,max_ value (Table 2), although it was at the lung/muscle tissue interface (cardiac-centred) and skin (brain-centred) tissue for both the CP mode and SAR-optimized RF phases. In the adult head, the SAR_10g,max_ value was not significantly improved (Table 2).

The whole-body and head averaged SAR values, were lower for the 8Tx-dipole array compared to the birdcage coil, although the differences were less pronounced in neonatal cardiac configuration (Table 2).

**Fig. 6:**
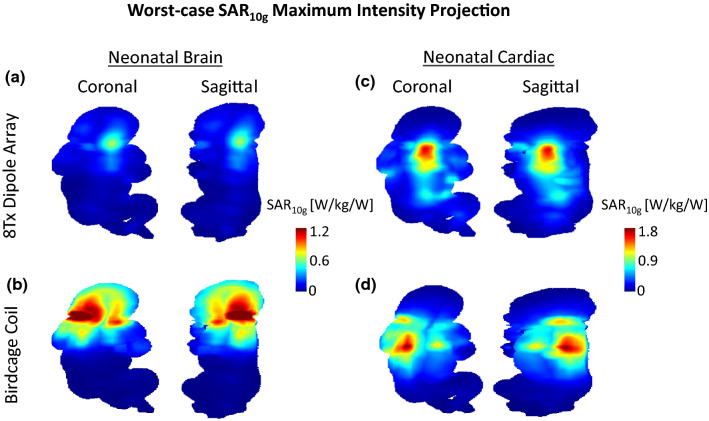
Maximum intensity projection worst-case SAR_10g_ maps shown for the 8Tx-dipole array and the birdcage coil in **a** and **b** the neonatal brain and **c** and **d** the neonatal cardiac positions

A worst-case local SAR (SAR_10g,wc_) analysis was performed for the 8Tx-dipole array to determine the RF weights producing the maximum power deposition levels in neonatal brain and cardiac setups. The highest SAR_10g_ intensity was found in muscle, at the neck level, as shown in the SAR_10g,wc_ map given for the neonatal brain configuration (Fig. 6a). The peak SAR_10g_ location moved towards the body’s centre at the lungs (throat is defined as inflated lungs) and muscle tissue interface in the cardiac position (Fig. 6c). The SAR_10g,wc_ peak value was increased from 0.65 W/kg/W in brain-centred to 1.61 W/kg/W in heart-centred positions but was significantly higher compared to the levels achieved in regular scenarios, for example for neonatal brain imaging, as demonstrated with the SAR-optimized RF shims (Table 2). The SAR levels achieved in the worst-case scenario for the 8Tx-dipole array were lower by 61% in the brain and 11% in cardiac neonatal setups in comparison with the birdcage coil. With the birdcage coil, the maximum SAR_10g_ was observed in skin and muscle at the neck level in the neonatal brain configuration (Fig. 6b) and at the lung/muscle tissue interface in the neonatal cardiac setup (Fig. 6d).

**Fig. 7:**
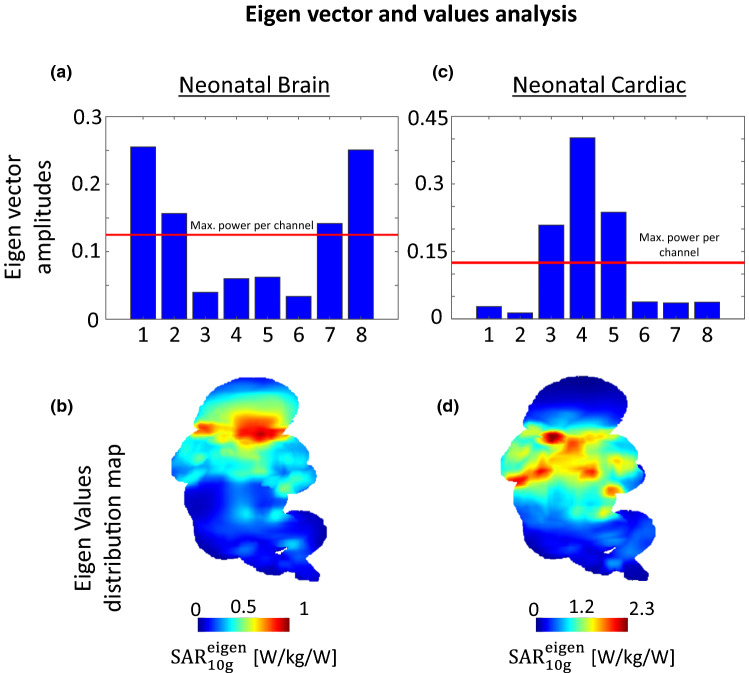
Eigen vector amplitudes corresponding to the maximum eigenvalue given per channel in **a** neonatal brain, and **c** neonatal cardiac configurations, normalized to 1 W input power for the 8Tx-dipole array. **b** and **d** Maximum intensity projection eigen-value maps shown for the two configurations

Dipoles 1 and 8 demonstrated the major contribution to the ${\mathrm{SAR}}_{10\mathrm{g},\mathrm{max}}^{\mathrm{eigen}}$ value in the neonatal brain configuration (Fig. 7a) while that were dipoles 3, 4 and 5 in the neonatal cardiac configuration (Fig. 7c). Dipole 6 only marginally contributed to the ${\mathrm{SAR}}_{10\mathrm{g},\mathrm{max}}^{\mathrm{eigen}}$ in both neonatal configurations (Fig. 7a–c). The position of the ${\mathrm{SAR}}_{10\mathrm{g},\mathrm{max}}^{\mathrm{eigen}}$ value was consistent with the SAR_10g,wc_ position (Fig. 7b–d). In the adult brain, the ${\mathrm{SAR}}_{10\mathrm{g},\mathrm{max}}^{\mathrm{eigen}}$ value was found when the dipole 8 gets almost all the available power.

**Fig. 8:**
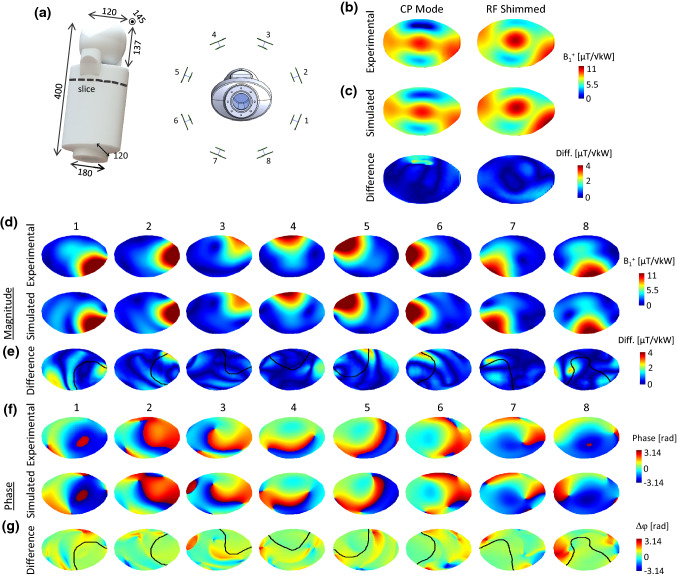
**a** CAD model of the neonatal phantom with general dimensions (in mm) and placement inside the 8Tx-dipole array. **b** and **d** Experimentally measured and simulated B_1_^+^-field maps, normalized to 1 kW total input power, shown in **b** for the CP mode and one RF shim configuration ([155 200 328 0 0 7 83 132] degrees), and in d) for individual transmit elements. **f** Experimentally measured and simulated individual phase maps corresponding to the transmit elements shown in **d**. The phase maps were computed relative to the shimmed mode. The slice position for all the results shown is indicated in **a**. **c**, **e**, **g** Difference maps calculated for the combined maps (**c**) and individual B_1_^+^-field maps (**e**, **g**). In **e** a contour line was drawn (in white) to visually represents the extent of the measured individual B_1_^+^-field distribution patterns (shown in d). In **e** and **g** a contour line was drawn (in black) to visually represents the extent of the measured individual B_1_^+^-field distribution patterns (shown in **d** and **f**). Note that the colour bar for the magnitude differences (**c** and **e**) have been rescaled to show residuals more clearly

Figure 8b shows the simulated and measured B_1_^+^-field maps for CP mode and one RF-shimmed configuration. The normalized root-mean square error (NRMSE) between simulated and measured B_1_^+^-field maps was about 6.3% in CP mode and 5.9% in RF shimmed case (Fig. 8c). Figure 8d, f shows the individual measured and simulated B_1_^+^-field maps, for both magnitude and phase. The NRMSE values for individual B_1_^+^-field magnitude maps ranged from 5.3% for channel 2–7.3% for channels 3 and 8 (Fig. 8e). The averaged phase differences between simulated and measured individual phase maps ranged from 3.7 degrees for channel 2 to 23.5 degrees for channel 8 (Fig. 8g). The contour lines (Fig. 8e and g, black line) demonstrate that the large differences in magnitude or phase happen outside the high B_1_^+^-field areas for individual channels.

## Discussions

In this study, an 8Tx-dipole array was designed and constructed towards neonatal brain and cardiac MR applications and evaluated through electromagnetic field simulations. The effects of subject size (baby vs adult) and target size (brain vs heart) on B_1_^+^-field efficiency and SAR levels were investigated in simulation, for the proposed dipole array, in contrast with a standard birdcage coil.

The dipole array demonstrated robustness against load-variation when the baby model was moved from the brain- to heart-centred positions with only small variation in reflection coefficients which remained better than -10 dB for all position tested. This suggests that the coil array could be used to image the brain or heart during a single MR session with minimal impact on the baby’s comfort. The birdcage coil demonstrated comparable results.

The SAR efficiency maps (${\mathrm{B}}_{1}^{+}/\sqrt{{\mathrm{SAR}}_{10\mathrm{g},\mathrm{max}}}$) demonstrated substantially improved performance for the 8Tx-dipole array in comparison with the birdcage coil and the 8Tx-loop coil array (Supporting Information Figure S1). This improvement is important for MR examinations as the SAR levels are a limiting factor at 7 T, and RF safety is a major concern for neonatal applications. In the adult brain, similar SAR efficiency levels were achieved compared to the neonatal brain configuration with the 8Tx-dipole array. Thus, although the body size difference from adult to baby’s head may contribute to improve the RF performance of the coil, the differences in dielectric properties strongly mitigate the potential improvements. This effect was more pronounced with the birdcage coil, with a 18% decreased SAR efficiency in neonatal brain configuration compared to the adult brain. This observation is consistent with the work presented by Malik et al. [49].

It is important to note that the neonatal results for the 8Tx-dipole array were achieved by optimizing the RF phases, which decreased the peak SAR_10g_ value with no drop of the averaged B_1_^+^-field value over the region-of-interest. However, when using the SAR-optimization cost function (Eq. 1), a certain bias is introduced during the computation. Indeed, the (SAR_10g,max_)_VOPs_ value overestimates the SAR_10g,max_ value obtained with the full Q-matrices by a variable coefficient, notably for low values [45]. Therefore, some solutions that may provide a low SAR_10g,max_ value are discarded by the algorithm. Nevertheless, the obtained results enhance the potential of parallel-transmit methods to improve RF safety of subjects, particularly neonates. Although a fixed CP mode produces an efficient B_1_^+^-field, the power deposition levels cannot be minimized. With independent optimization of the RF weights, lower SAR_10g,max_ values could be achieved. In CP mode, the SAR_10g,max_ position was outside of the neonatal brain with the 8Tx-dipole array. Therefore, the algorithm can alleviate the SAR levels located outside of the brain without compromising the B_1_^+^-field efficiency inside the brain. However, B_1_^+^-field levels are likely to be decreased in the other regions.

The lower power deposition levels obtained with the 8Tx-dipole array compared to the birdcage coil were also observed in terms of whole-body and head-average SAR values for neonate. The increased number of transmit elements and subsequently the higher coupling levels, may be responsible for these differences. In addition, unlike the birdcage coil, the 8Tx-dipole array was not shielded, which may have led increased radiation losses. For the 8Tx-dipole array, the absorbed power in the tissue was 71% (brain-centred) and 27% (cardiac-centred) lower compared to the birdcage coil for the SAR-optimized situations.

The IEC guidelines provide the safe operation limits for RF coils used in MRI [46], in which the ratio between the maximum authorized SAR_10g_ value (10 W/kg) and the head-average (3.2 W/kg) or whole-body average (2 W/kg) SAR is 3.1 and 5, respectively. If the ratio of the predicted SAR_10g_ to either the head-average or whole-body average SAR is smaller than the ratio of the respective limits, this indicates that the relevant average SAR value will be the limiting factor. In neonatal cardiac configuration, the SAR_10g,max_ is the limiting quantity, except for the SAR optimized case, while in the brain configuration, it is either whole-body or head-average SAR for the 8Tx dipole array. For the birdcage coil, the SAR_10g,max_ is always the limiting quantity. These two quantities should be further investigated for safe neonate imaging at 7 T. The SAR-optimized cost function efficiently decreased the peak SAR_10g,max_ value, but has only little effect on the overall power deposited.

The worst-case SAR_10g_ analysis demonstrated that the maximum SAR_10g_ value was significantly increased from the neonatal brain-centred to cardiac-centred setups. However, in the MR system, the RF safety limits are usually normalized to the absorbed power in the tissue. Doing so, the relative difference is about 2.3%. This observation supports the feasibility of neonatal brain and cardiac imaging without modifying the RF safety power limits, would the worst-case SAR values be used. The same observation applies for the birdcage coil. The scanner RF power limits could be dynamically adjusted regarding the applied RF phases in the case of the 8Tx-dipole array, instead of worst-case scenario. In this way, more flexibility would be granted in terms of MR protocols and scan time. To allow this, a combined VOP set could be prepared accounting for both the neonatal brain and cardiac cases. Although RF phase optimization demonstrated the ability to reduce the local SAR_10g_, other parallel-transmit approaches as pulse optimization could be used to decrease SAR_10g_ levels with the 8Tx-dipole array [50–53].

A good agreement between the simulated and measured B_1_^+^-field magnitude and phase maps (Fig. 8) was shown both qualitatively and quantitatively and represents an important step for validation of the simulated SAR model. The worst-case SAR limits should be used in case there is a mismatch between simulated and measured data for the individual phase maps to avoid power effectively deposited in tissues being different from the simulated one when RF shimming is used. However, significant additional validation data are still required while approaching to use phase-specific limits, such as thermometry measurements [54].

For shimmed RF phases, the SAR_10g,max_ location is mainly driven by interferences between all the individual RF fields, but the dipoles presenting the higher contribution to the ${\mathrm{SAR}}_{10\mathrm{g},\mathrm{max}}^{\mathrm{eigen}}$ may correspond to the most SAR-sensitive elements. The dipole design at these positions could be further investigated to decrease the individual SAR_10g_ levels. It is interesting to note that while for the adult head, a single dipole is responsible for the highest SAR_10g_ level, for the neonate body the trend is more unclear. Therefore, although dipole design can be improved, replacing only one or two dipoles may not be beneficial.

Considering the results obtained, it appears that the smaller subject size (baby vs adult) resulted in enhanced B_1_^+^-field, although the distance to dipole elements was large. With respect to the target size and location (brain vs heart), the results demonstrate that SAR efficiency levels are slightly better in cardiac configuration compared to brain.

Nevertheless, although the 8Tx-dipole array performed well for neonatal brain and cardiac applications in terms of SAR efficiency, the large distance and the small number of dipoles may significantly limit the receive performance, notably for signal-to-noise (SNR) ratio and acceleration capabilities. At 3 T, building dedicated receive arrays for neonatal brain demonstrated significant SNR improvements compared to adult coils [55–57]. Further developments will, therefore, include the design and construction of a multi-channel receive array insert optimized for spatial coverage from the neonatal brain to the heart at 7 T.

## Conclusion

An 8Tx-dipole array body coil format for neonatal brain and cardiac MR was successfully demonstrated with both brain and heart covered in electromagnetic field simulations. Building a dedicated coil allows adjusting the design with respect to the very specific needs for neonate imaging in terms of positioning and comfort. The parallel-transmit approach for neonate imaging outperformed the single-transmit approach with the capability to tackle the SAR_10g,max_ value while keeping the benefit of increased B_1_^+^-field efficiency. We conclude that the 8Tx-dipole array promises safe operating procedures for MR imaging of neonatal brain and heart at 7 T.

## Supplementary Information

Below is the link to the electronic supplementary material.

Supplementary file1 (STEP 16229 kb)

Supplementary file2 (PDF 348 kb)
