# Supplementary material for: Towards an integrated neonatal brain and cardiac examination capability at 7 T: electromagnetic field simulations and early phantom experiments using an 8-channel dipole array
Source: MAGMA. 2022 Jan 8;35(5):765–78. doi: 10.1007/s10334-021-00988-z (PMC9463228; doi:10.1007/s10334-021-00988-z)
Supplement: Supplementary file 2 — Supplementary file2 (PDF 348 kb) [file 10334_2021_988_MOESM2_ESM.pdf]

Supporting Information

As a comparison, an 8Tx-loop coil array was modelled and simulated in both neonatal configurations. In the following paragraphs, we briefly describe the simulation model and the results obtained.

Methods

Eight loops (230 mm x 80 mm) were designed with identical longitudinal extent and placed at same distance from the load and position compared to the 8Tx-dipole array using the CAD model of the coil array frame (Supporting Figure S1a). The 8Tx-loop coil array also included the FR-4 substrate ($\varepsilon_r = 4$, zero electrical conductivity). To decouple the nearest neighbour loop coils, decoupling capacitors (Supporting Figure S1a, blue lines) were placed in-between the loops following the approach described by Yan et al. [1]. However, since the coil array is splittable, no decoupling capacitors were placed between loops 1 and 2 and 5 and 6. The values of decoupling capacitors were optimized in co-simulation for nearest neighbour decoupling, and the loop coils were tuned and matched to 297.2 MHz and 50 Ohms in the neonatal brain configuration. The same lumped element values were applied in the neonatal cardiac position and simulated S-matrices were computed. RF phases were optimized using the SAR-optimized cost function (Equation 1), and SAR efficiency maps ($B_1^+/\sqrt{SAR_{10g,max}}$) were calculated and compared to the 8Tx-dipole array (Supporting Figure S1d).

Results

The 8Tx-loop coil array achieved $S_{i,i}$ values better than -17 dB, an averaged $S_{i,j}$ value of -9.2 dB and $S_{i,j+1}$ values ranging from -13.5 dB to -4.8 dB in the neonatal brain and cardiac configurations (Supporting Figure S1b). Averaged SAR efficiency values in the brain and heart were, respectively, 23% and 18% lower compared to the 8Tx-dipole array (Supporting Figure S1c-d).

Discussion and conclusion

An 8Tx-loop coil array was simulated following identical design criteria as for the 8Tx-dipole array. The SAR efficiency levels were higher for the 8Tx-dipole array compared to the 8Tx-loop array in both neonatal configurations. Nearest-neighbour decoupling was achieved, but the next-neighbour coupling levels were significantly high. We conclude that, given the design criteria and coil configuration, the 8Tx-dipole array is a more valuable approach for neonatal brain and cardiac MR than the 8Tx-loop coil array.

8Tx-loop coil array results

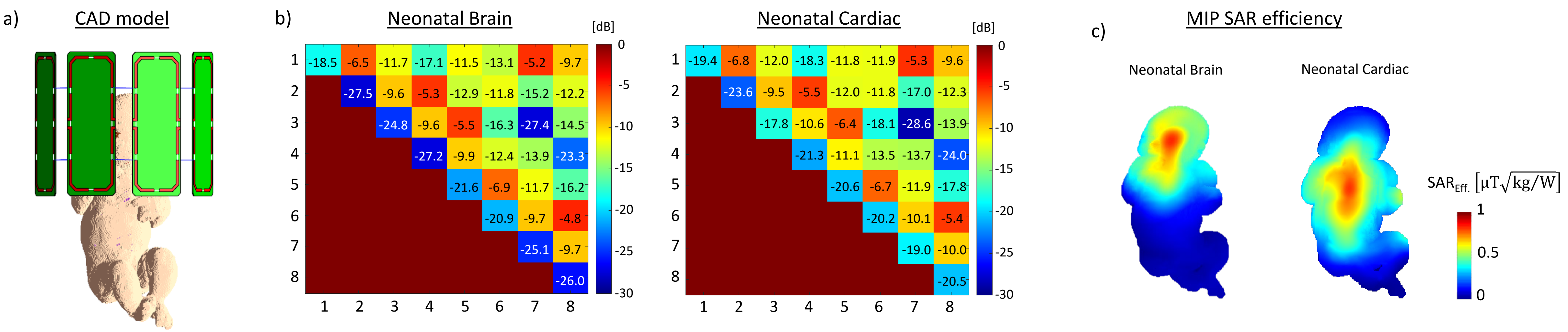

d)

	Coil Model	Mean B_1^+ over ROIs $[\mu T/\sqrt{W}]$	SAR efficiency $[\mu T\sqrt{kg/W}]$	$SAR_{10g,max}$ $[W/kg/W]$	Whole-body average SAR $[W/kg/W]$	Head-average SAR $[W/kg/W]$	Absorbed Power [%]
Neonatal Brain	8Tx Dipole Array	0.32 ± 0.06	0.64 ± 0.12	0.25	0.07	0.13	24
	8Tx Loop Array	0.28 ± 0.06	0.49 ± 0.10	0.33	0.05	0.12	19
Neonatal Cardiac	8Tx Dipole Array	0.61 ± 0.11	0.73 ± 0.13	0.69	0.18	0.16	66
	8Tx Loop Array	0.39 ± 0.09	0.60 ± 0.14	0.42	0.09	0.08	33

Supporting Figure S1. a) Simulated CAD design of the 8Tx-loop coil array and the neonate model. b) Simulated S-matrices shown in the neonatal brain and cardiac configurations. The loop coils were tuned and matched in neonatal brain configuration, and the same lumped element values were used in the cardiac configuration. c) Maximum intensity projection of SAR efficiency ($B_1^+/\sqrt{SAR_{10g,max}}$) maps, shown in coronal orientation for the 8Tx-loop coil array in the neonatal brain and cardiac configurations. RF phases were optimized over a ROI in a slice for $SAR_{10g,max}$ reduction. d) Quantitative results of the maps shown in c). The results obtained with the 8Tx-dipole array (Table 2) are given for comparison. All the quantities were normalized to 1W input power.
